# Antimicrobial use and mortality among intensive care unit patients with bloodstream infections: implications for stewardship programs

**DOI:** 10.1016/j.heliyon.2022.e10076

**Published:** 2022-08-04

**Authors:** Mera A. Ababneh, Mohammad Al Domi, Abeer M. Rababa'h

**Affiliations:** Department of Clinical Pharmacy, Faculty of Pharmacy, Jordan University of Science and Technology, Irbid, Jordan

**Keywords:** ICU, Mortality, Bloodstream infection, Antibiotic use

## Abstract

**Background:**

Bloodstream infections (BSIs) are one of the most critical illnesses requiring intensive care unit (ICU) admission. Antimicrobial therapy (AMT) is one of the vital management strategies for the treatment of BSIs; it should be chosen appropriately to reduce mortality.

**Objectives:**

This is the first study to investigate the types of antimicrobial agents administered in the ICU setting and the predictor variables associated with mortality.

**Methods:**

This retrospective study was conducted at King Abdullah University Hospital (KAUH). All hospitalized patients admitted to the ICU and received at least one antimicrobial agent over 3 years period (January 1, 2017, to December 31, 2019) were included in the study. Electronic patients' medical records were used to collect patients' demographic and clinical characteristics, patient general health status, events that occurred during hospitalization, and events after obtaining the blood culture. Descriptive analysis was done to identify the types of antimicrobials used and the distribution of the microorganisms among the study participants. The susceptibility test of the bloodstream culture was checked for each patient**.** Moreover, crude mortality and its associated factors were investigated.

**Results:**

A total of 1051 patients were enrolled in the study, where 650 patients (61.84%) were treated with three or more antimicrobial agents. The most frequent antimicrobials used were piperacillin/tazobactam followed by teicoplanin, meropenem, and levofloxacin. About half of the patients died within 30-days of BSI, which was associated with several factors including advanced age, presence of co-morbidities, nosocomial infections or healthcare-associated infections, length of ICU stay, respiratory tract infections, receiving vasopressor during the hospital stay, concurrent positive culture other than blood with BSI, receiving combination antimicrobial therapy, those who were complicated with septic shock or renal failure, receiving total parenteral protein (TPN) nutrition, and inappropriate empiric antimicrobial therapy.

**Conclusion:**

In conclusion, the administration of the antimicrobials among ICU patients was highly based on a combination of three or more agents covering a broad spectrum of microorganisms. The mortality rate was high among patients which were associated with inappropriate empirical therapy. Therefore, the antimicrobial stewardship (ASP) protocol has to be evaluated in the hospital for ICU patients. Moreover, we suggest recommending that hospital policies should apply the ASP protocol, infection control, implement the antimicrobial de-escalation protocol, and do best controlling on the co-morbid conditions, especially for ages 65 years or more to reduce the mortality rate in the ICU.

## 1 Introduction

Critical illness is a life-threatening condition that requires admission to the intensive care unit (ICU). Patients in the ICU are at higher risk for mortality and morbidity. Therefore, they require special health care management to improve their health outcomes and optimize their quality of life.

Several conditions were associated with critical illness including severe trauma, the postsurgical state, pancreatitis, burn injury, hemorrhage, ischemia, and infections or sepsis [1].

Sepsis is an infection of the blood that results in life-threatening organ dysfunction [2], primarily caused by bacterial pathogens [3, 4]. Several risk factors were associated with the development of sepsis such as the severity of illness, disruption of anatomical barriers, impaired immunological response [5], as well as exposure to several types of procedures such as intubation, mechanical ventilation, and vascular access.

Administration of antimicrobial therapy (AMT) is one of the management strategies needed for ICU patients with sepsis. AMT should be selected appropriately; because ineffective or inappropriate AMT will lead to harmful outcomes, including the development of multidrug-resistant (MDR) organisms which are associated with a longer hospital stay, longer ICU stay, and higher mortality rates [6, 7, 8, 9, 10]. Broad-spectrum antimicrobials are not necessary to be administered for all patients; selected patients may require this extended coverage of antimicrobials, including multi-organ failure, invasive catheters, previous healthcare exposure, antibiotic use, and immunosuppression [6, 11]. Consequently, the appropriate selection of empiric antimicrobial therapy should be based on the patient's specific factors and the location source of the infection.

ICU patients usually present higher mortality rates due to infections; a retrospective observational study conducted at the ICU of the King Fahad Hospital, Jeddah, Saudi Arabia, 39 out of 52 infectious patients died with a case-fatality rate of 75% [12]. Another retrospective study was conducted in the Cardiac Surgical Intensive Care Unit demonstrated that mortality risks were statistically significantly different between the groups with and without nosocomial infections (NI) (P < 0.001) [13]. The overall case-fatality rate associated with bloodstream infections (BSIs) was 15%–20% and 35%–50% when patients with ICU admission are considered [5]. A multi-center study was conducted to investigate the outcomes of infected ICU patients, showing that the infected patients had significantly higher ICU and hospital mortality rates and longer ICU and hospital lengths of stay when compared to those who did not have infections [14].

Due to limited studies evaluating the antimicrobial use and resistance in Jordan, this study was conducted and aimed to assess the types of antimicrobial agents used among ICU patients with sepsis as well as the incidence and predictors of mortality among ICU patients with BSI.

## 2 Methods

### 2.1 Study design and setting

This retrospective study was conducted among ICU patients who were administered at least one antimicrobial agent from January 1, 2017, to December 31, 2019. The study was conducted at KAUH, a tertiary care hospital in Jordan. The study was approved by the Institutional Review Board (IRB) at KAUH.

### 2.2 Data collection

Electronic patients' medical records and charts in KAUH were used to obtain demographic and clinical information for each patient. Any patient who presents with multiple episodes of ICU admission within one year period was included as a single participant using the first episode, and other episodes were excluded. In addition, patients with incomplete information in their medical records and charts were excluded from the study.

The demographic and clinical information is composed of four parts:

Part 1 Patient demographic characteristics (age, gender, weight, height, BMI, length of hospital stay, length of ICU stay).
Part 2 Patient general health status (smoking, the presence of co-morbidities such as (hypertension, diabetes mellitus, myocardial infarction, atrial fibrillation, congestive heart failure, pulmonary diseases, chronic kidney disease, end-stage renal disease, cerebrovascular disease, solid tumors, lymphoma, leukemia, dementia, and prior major surgery), recent invasive procedure within 48 h of admission such as (bronchoscopy, central venous catheter, chest tubes, surgery, mechanical ventilation, central arterial catheter, folly's catheter, and pig tubes), previous hospitalization within 90 days of positive blood culture, transferring from another hospital to KAUH and previous antimicrobials administered within 90 days before hospital admission).
Part 3 Events that occurred during hospitalization (the primary ward/unit admission, administration of Vasopressors, blood transfusion, type of nutrition support, pathogens isolated from body sites other than blood, and antimicrobials that were given during ICU stay).
Part 4 Events that occurred after obtaining the blood culture (empiric antimicrobials, definitive antimicrobials, pathogens were obtained from a blood test, the sensitivity test results, the mortality within 14-day and 30-day of positive blood culture, and the complications of the infection).

Susceptibility test was checked for each patient and crude mortality was calculated, and appropriate tests were conducted to assess mortality risk factors.

### 2.3 Clinical outcome measures

The first outcome was the types of antimicrobial agents used and the distribution of microorganisms in the ICU.

The second outcome was 14-day and 30-day mortality as well as the independent variables that are associated with mortality.

### 2.4 Definitions

BSIs were defined as positive blood cultures with simultaneous signs and symptoms of infection. Among the clinical outcomes of the study, any antimicrobial that was given in the period between collecting the blood sample and obtaining the susceptibility test result was considered empiric therapy. On the other hand, any antimicrobial prescribed after obtaining the result of the susceptibility test was considered definitive therapy. To assess the appropriateness of empiric therapy, two main points must be met: the empiric drug therapy was given within 24 h of blood sample collection PLUS the infecting pathogen is sensitive to at least one of the given antimicrobial agents according to the susceptibility test results. Similarly, definitive therapy deems appropriate if it fulfilled two criteria: prescribed within 24 h of the susceptibility test results PLUS the infecting pathogen is sensitive to at least one of the administered antimicrobial agents according to the susceptibility test results. 14-day and 30-day mortality are defined as death within 14-day and 30-day of the first positive blood culture, respectively.

### 2.5 Microbiology testing

The VITEK II system (bioMerieux, Balmes-Les-Grottes, France) identified the isolates during the study period. Antimicrobial susceptibility testing was performed by the microdilution method on the VITEK II system. The Clinical and Lab Standard Institute (CLSI) breakpoints were used to determine the susceptibility to the antimicrobial agents tested for the study period, as reported by the microbiology laboratory.

## Ethical approval

The institutional review board at King Abdullah University Hospital (KAUH) approved the study and informed consent was waived.

### 2.6 Statistical analysis

Statistical analysis was performed using SPSS (Statistical Package for the Social Sciences) version 23. Descriptive analysis was presented as the mean and standard deviation for continuous data, whereas frequencies and percentages were used to summarize categorical data. To identify independently associated with mortality at 14- and 30-day, a multivariate forward, stepwise logistic regression analysis was performed with p < 0.10 to stay and p < 0.05 to report. This was preceded by conducting univariate analysis to determine variables to be included in the multivariable model. Odds ratio (OR) with 95% confidence interval (CI) were calculated. All tests performed were 2-tailed tests of significance and a p-value less than 0.05 was considered significant.

## 3 Results

### 3.1 Demographic and clinical characteristics

A total of 1051 patients were enrolled in this study; all of them had received at least one antimicrobial agent and were admitted to the ICU during the study period.

The demographic and clinical characteristics are presented in Table 1. The mean age of study participants was 60.2 ± 19.3, 54.5% were males and 88.4% were non-smokers. . The distribution of morbidities among study participants was as follows; known cases of hypertension (HTN) (55.6%) and diabetes mellitus (DM) (45.8%), admitted to the ICU (86.4%) as primary ward admission with nosocomial infection (51.2%) of a respiratory focus site (34.3%). In addition, 36.1% and 22.0% of the study participants were hospitalized within 90 days before admission and transferred to King Abdullah University Hospital (KAUH), respectively. Only 185 patients underwent invasive procedures within 48 h before admission.

**Table 1:** Demographic and clinical characteristics of 1051 patients admitted to ICU.

Characteristics	Mean ± SD	Number (%)
**Age (years)**	60.2 ± 19.3	
**BMI (kg/m**^**2**^**)**	27.6 ± 7.2	
**Gender**		
Male Female		573 (54.5%) 478 (45.5%)
**Smoking**		
Smoker Not smoker		174 (16.6%) 877 (83.4%)
**Co-morbidities**		
Hypertension Diabetes mellitus Myocardial infarction Atrial fibrillation Congestive heart failure Pulmonary disorders Chronic kidney disease End-stage renal disease Cerebrovascular disease Solid tumor Lymphoma Leukemia Dementia Previous surgery Others		584 (55.6%) 481 (45.8%) 176 (16.8%) 67 (6.4%) 119 (11.3%) 64 (6.1%) 81 (7.7%) 74 (7.0%) 151 (14.4%) 154 (14.7%) 14 (1.3%) 12 (1.2%) 11 (1.01%) 52 (5.0%) 360 (34.3%)
**Invasive procedures within 48 h of admission**		
Bronchoscopy Venous catheter Chest tube Arterial catheter Mechanical ventilation Foleys catheter Pig tube		3 (0.3%) 9 (0.9%) 6 (0.6%) 11 (1.1%) 84 (8.0%) 55 (5.2%) 17 (1.6%)
**Previous hospitalization (within 90 days)**		
Yes No		379 (36.1%) 672 (63.9%)
**Hospital transfer**		
Yes No		231 (22.0%) 820 (78.0%)
**Acquisition site**		
Nosocomial Healthcare-associated Community-acquired		538 (51.2%) 141 (13.4%) 372 (35.4%)
**Infection focus site**		
Respiratory Genitourinary Line-related Gastrointestinal Biliary S CNS Unknown		360 (34.3%) 191 (18.2%) 47 (4.5%) 80 (7.6%) 5 (0.5%) 69 (6.6%) 68 (6.5%) 231 (22.0%)
**Primary ward admission**		
ICU Surgery Medical CCU Oncology		908 (86.4%) 47 (4.5%) 73 (7.0%) 14 (1.3%) 9 (0.9%)

SSTI = Skin and soft tissue infection. CNS = Central nervous system. ICU = Intensive care unit. CCU = coronary care unit.

### 3.2 The pattern of antimicrobial use and distribution of microorganisms

During ICU stay, a total of 153 patients (14.6%) were treated with antimicrobial monotherapy, 248 patients (23.60%) were treated with dual antimicrobial therapy, whereas most of the patients (650 patients; 61.8%) were treated with three or more antimicrobial agents.

The patterns of antimicrobial use during ICU stay are illustrated in Table 2. During ICU stay, patients were treated, predominately, with glycopeptides (69.9%), piperacillin/tazobactam (65.0%), carbapenems (63.0%), fluoroquinolones (40.9%), cephalosporins (30.4%), and aminoglycosides (15.6%) were also commonly used. Only 255 patients out of 1051 (24.3%) had received antifungal agents, primarily fluconazole (15.4%).

**Table 2:** The pattern of antimicrobial use during ICU stay for 1051 patients.

Antimicrobial agents	Number (%)
**Glycopeptides**	735 (69.9%)
Teicoplanin Vancomycin	466 (44.3%) 269 (25.6%)
**Penicillins**	683 (65.0%)
Piperacillin/tazobactam Amoxicillin/clavulanic acid Ampicillin Amoxicillin	653 (62.1%) 19 (1.8%) 8 (0.8%) 3 (0.3%)
**Carbapenems**	662 (63.0%)
Meropenem Imipenem Ertapenem	425 (40.4%) 222 (21.1%) 15 (1.4%)
**Fluoroquinolone**	430 (40.9%)
Levofloxacin Ciprofloxacin	348 (33.1%) 82 (7.8%)
**Cephalosporins**	319 (30.4%)
Cefazoline Ceftriaxone Cefuroxime Cefixime Cefotaxime	150 (14.3%) 146 (13.9%) 17 (1.6%) 3 (0.3%) 3 (0.3%)
**Aminoglycosides**	164 (15.6%)
Gentamycin Amikacin Tobramycin	100 (9.5%) 63 (6.0%) 1 (0.1%)
**Oxazolidinone**	37 (3.5%)
Linezolid	37 (3.5%)
**Glycylcyclin**	14 (1.3%)
Tigecycline	14 (1.3%)
**Macrolides**	10 (0.9%)
Clarithromycin Azithromycin Erythromycin	8 (0.8%) 1 (0.1%) 1 (0.1%)
**Tetracyclines**	7 (0.7%)
Doxycycline	7 (0.7%)
**Miscellaneous**	285 (27.1%)
Colistin Metronidazole TMP/SMX Rifampin	145 (13.8%) 120 (11.4%) 15 (1.4%) 5 (0.5%)
**Anti-fungal agents**	255 (24.3%)
Fluconazole Caspofungin Anidulafungin Nystatin Voriconazole Amphotracin B	162 (15.4%) 41 (3.9%) 39 (3.7%) 36 (3.4%) 11 (1.1%) 2 (0.2%)

TMP/SMX = Trimethoprim-Sulfamethoxazole.

There were a total of 44 different types of pathogens were isolated from the study patients, half of them (50%) were gram-negative bacteria, only 14 out of 44 (31.8%) were gram-positive bacteria and only eight pathogens were fungi (18.2%). The susceptibility test was done for 39 types of pathogens which resulted in a total of 442 susceptibility tests. These susceptibility tests were conducted for only 378 patients (out of 1051; 36.0%). Most of our study participants were infected with only one pathogen (882 out of 1051; 83.9%), whereas the rest were infected with polymicrobial pathogens as follow: 132 patients (12.6%) were infected with two pathogens, 31 patients (2.9%) were infected with three pathogens, four patients (0.4%) were infected with four pathogens, and only two patients (0.2%) were infected with five pathogens. Because of polymicrobial infections; the total positive blood culture results are where equal to 1265.

**Figure 1:**
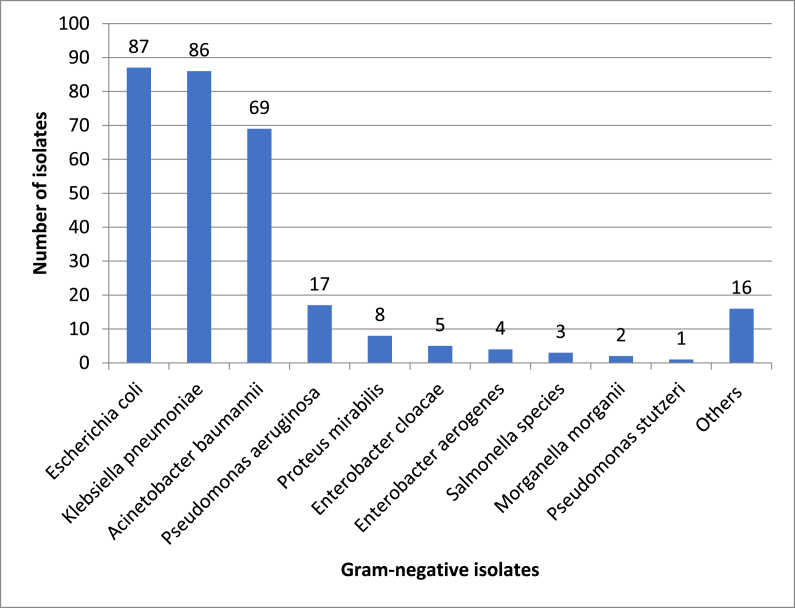
Frequency and species distribution of 298 Gram-negative bacteria isolates.

**Figure 2:**
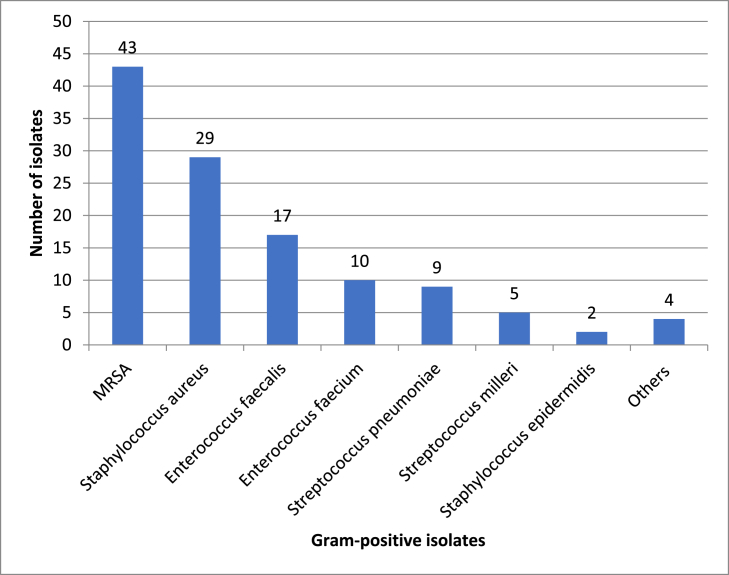
Frequency and species distribution of 119 Gram-positive isolates.

The type of pathogens identified by the microbiology database for study participants is summarized in Figures 1 and 2. Our patients were infected more frequently with gram-negative bacteria compared to gram-positive bacteria and fungi. The predominant gram-negative bacteria that infected our study participants were *Escherichia coli*, followed by *Klebsiella pneumonia, Acinetobacter baumannii, and Pseudomonas aeruginosa.* On the other hand, the most predominant gram-positive bacteria were *MRSA,* followed by *Staphylococcus aureus, Enterococcus faecalis*, *Enterococcus faecium*, and *Streptococcus pneumonia*. *Regarding fungal infections,* they account for the lowest percentage compared to bacterial infections (6.6%). As mentioned previously, susceptibility tests were conducted for 378 patients, a total of 273 patients of them (72.22%) were shown to be infected with MDR pathogens (resistant to at least three antimicrobial classes). The most frequently MDR pathogens infected our study patients were *Acinetobacter baumannii* was 67/69 (97.10%), E-coli was 76/87 (87.36%), *MRSA* was 37/43 (86.04%), *Klebsiella pneumonia* was 73/86 (84.88%), *Pseudomonas aeruginosa* was 10/17 (58.83%), *Enterococcus species* 12/27 (44.44%), *Streptococcus pneumonia* 3/9 (33.33%), *Candida albicans* was 2/6 (33.33%), *Staphylococcus aureus (MSSA)* 4/29 (13.79%).

### 3.3 Mortality and associated factors

About half of the patients (47.9%) have died within 14-days of the first positive blood culture. Based on the univariate analysis, multiple factors were associated with 14-day mortality as depicted in Table 3. Older adults had a higher risk of mortality compared to the younger age group.

**Table 3:** Univariate analysis of the risk factors associated with 14-day mortality in ICU patients with BSI.

Variable	Died N = 503,(%)	Survived N = 548, (%)	Odd ratio	95% CI	P-value
Gender					
Female Male	222 (44.1%) 281 (55.9%)	256 (46.7%) 292 (53.3%)	0.92	0.72–1.18	0.4986
Age group					
18–64 65 and more	224 (44.5%) 279 (55.5%)	335 (61.1%) 213 (38.9%)	1.92	1.52–2.44	<0.0001∗
BMI					
<30 ≥30	231/332 (69.6%) 101/332 (30.4%)	297/441 (67.3%) 144/441 (32.7%)	0.93	0.69–1.26	0.6508
Smoking					
Yes	76 (15.1%)	98 (17.9%)	0.74	0.54–1.01	0.0631
Co-morbidities					
DM CKD HTN Pulmonary disease ESRD Solid tumor CVD MI AF CHF	239 (47.5%) 49 (9.7%) 310 (61.6%) 38 (7.6%) 26 (5.2%) 108 (21.5%) 82 (16.3%) 94 (18.7%) 36 (7.2%) 69 (13.7%)	242 (44.2%) 32 (5.8%) 274 (50.0%) 26 (4.7%) 48 (8.8%) 46 (8.4%) 69 (12.6%) 82 (15.0%) 31 (5.7%) 50 (9.1%)	1.13 1.66 1.53 1.47 1.62 3.01 1.33 1.17 1.18 1.57	0.89–1.43 1.0–2.43 1.21–1.94 0.91–2.4 1.01–2.63 2.18–4.44 0.96–1.86 0.86–1.60 0.73–1.93 1.08–2.27	0.3032 0.0478∗ 0.0004∗ 0.1182 0.0466∗ <0.0001∗ 0.0913 0.3037 0.4841 0.0172∗
Total co-morbidities					
0 1–4 ≥5	40 (8.0%) 403 (80.1%) 60 (11.9%)	104 (19.0%) 402 (73.4%) 42 (7.6%)	2.51 3.42	1.69–3.80 2.05–5.8	<0.0001∗ <0.0001∗
Mechanical ventilation					
Yes	40 (8.0%)	44 (8.0%)	1.07	0.69–1.67	0.7543
Prior catheterization					
Yes	42 (8.3%	50 (9.1%)	1.01	0.66–1.53	0.9561
Previous hospitalization (90 days hospitalization)					
Yes	190 (37.8%)	189 (34.5%)	1.15	0.90–1.47	0.2554
Hospital transfer					
Yes	90 (17.9%)	141 (25.7%)	0.65	0.48–0.86	0.0034∗
Acquisition site					
Nosocomial Healthcare-associated Community-acquired (R)	288 (57.3%) 59 (11.7%) 156 (31.0%)	250 (45.6%) 82 (15.0%) 216 (39.4%)	1.65 1.07	1.27–2.13 0.78–1.57	<0.0001∗ 0.7231
Source of infection					
Respiratory Genitourinary Line related GI SSTI CNS	205 (40.8%) 87 (17.3%) 13 (2.6%) 40 (8.0%) 17 (3.4%) 33 (6.6%)	155 (28.3%) 104 (19.0%) 34 (6.2%) 40 (7.3%) 52 (9.5%) 35 (6.4%)	1.66 0.92 0.49 1.03 0.32 0.97	1.30–2.13 0.69–1.25 0.2637–0.88 0.66–1.6 0.18–0.55 0.59–1.56	<0.0001∗ 0.6259 0.0213∗ 0.8920 <0.0001∗ 0.8918
Vasopressor use					
Yes	254 (50.5%)	142 (25.9%)	2.94	2.32–3.86	<0.0001∗
Blood transfusion					
Yes	49 (9.7%)	83 (15.1%)	0.62	0.42–0.89	0.0093∗
Pathogens from other body sites					
Yes	326 (64.8%)	328 (59.9%)	1.37	1.08–1.75	0.0101∗
Antimicrobial therapy					
Monotherapy (R) Dual-therapy Triple therapy or more	44 (8.7%) 119 (23.7%) 340 (67.6%)	109 (19.9%) 129 (23.5%) 310 (56.6%)	2.4 2.9	1.6–3.43 2.07–4.01	<0.0001∗ <0.0001∗
Septic shock					
Yes	204 (40.6%	76 (13.9%	4.44	3.32–6.00	<0.0001∗
Renal failure					
Yes	48 (9.5%)	28 (5.1%)	2.05	1.29–3.3	0.0022∗
TPN					
Yes	189 (37.6%)	144 (26.3%)	1.81	1.40–2.35	<0.0001∗
ICU stay (days)					
≤7 days >7 days	226 (44.9%) 277 (55.1%)	295 (53.8%) 253 (46.2%)	1.72	1.2–1.93	0.0004∗
Appropriate empiric					
Yes	82/174 (47.1%)	103/177 (58.2%)	0.6	0.43–0.92	0.0472∗
Appropriate definitive					
Yes	83/123 (67.5%)	142/182 (78.0%)	0.63	0.38–1.07	0.0877

BMI = Body mass index, DM = Diabetes mellitus, CKD = Chronic kidney disease, HTN = Hypertension, ESRD = End stage renal disease, CVD = Cerebro-vascular disease, MI = Myocardial infarction, AF = Atrial fibrillation, CHF = Congestive heart failure, GI = Gastro-intestinal, SSTI = Skin and soft tissue infection, CNS = Central venous system, TPN = Total parenteral nutrition, ICU = Intensive care unit.

(OR 1.92, p < 0.0001). Among the co-morbid diseases, chronic kidney disease (CKD), end-stage renal disease (ESRD), HTN, congestive heart failure (CHF), and solid tumors were associated with higher mortality. When comparing the types of infections, the mortality risk for patients infected with nosocomial infections was 1.65 times the risk of community-acquired infections (p < 0.0001). Bearing in mind that respiratory infection sources were associated with higher mortality risk but line-related or skin and soft tissue infections (SSTI) were observed to be associated with lower mortality (OR 0.49 and 0.32, respectively). When comparing patients with only BSI and those with (BSI and positive culture of body site other than blood), the latter group was at higher mortality risk (OR 1.37, p = 0.0101).

Moreover, the number of antimicrobial agents used to treat patients' infection was significantly associated with the mortality rate; patients who had dual and or 3 or more antimicrobial agents had higher rates of mortality (OR:2.4, p < .0001, OR: 2.9, p < 0.0001, respectively.). Other variables that were identified in the univariate analysis were vasopressor use during their hospital stay (OR 2.94, p < 0.0001), length of ICU (OR: 1.72; p = 0.0004). t renal failure (OR: 2.0; p = 0.0022), septic shock (OR: 4.5, p < 0.0001), patients on TPN (OR: 1.81, p < 0.0001). Nevertheless, several factors were significantly associated with lower mortality rates at 14 days, including hospital transfer (OR 0.65, p = 0.0034), blood transfusion during hospital stay (OR 0.62, p = 0.0093), and treatment with appropriate empiric antimicrobials (OR 0.60, p = 0.0472).

As shown in Table 4, the multivariable logistic regression showed that patients aged 65 years or more are significantly at high risk of death than younger patients after adjusting other factors (OR 2.79, p < 0.001). Also, patients with solid tumors and renal failures secondary to BSI are at higher mortality risk (OR 3.47, p < 0.001). In addition, those who were with nosocomial infections had a high mortality rate (p = 0.0262). On the other hand, patients treated with appropriate empiric therapy are at lower mortality risk when compared with inappropriate therapy (OR 0.56, p = 0.02).

**Table 4:** Multivariable analysis of the risk factors of 14-day mortality in ICU patients with BSIs.

Factor	Odd ratio	95% Confidence interval	P-value
Age group			
18–64 65 and more	2.79	1.76–4.53	<0.0001∗
Solid tumor	3.47	1.84–6.8	<0.0001∗
Acquisition site			
Nosocomial Healthcare-associated Community-acquired	1.82 0.69	1.07–3.11 0.35–1.37	0.0262∗ 0.2969
Septic Shock	3.87	2.28–6.4	<0.0001∗
Appriopaiate emorical therapy	0.56	0.35–0.91	0.02∗

Regarding mortality at 30-days, many of the risk factors identified for 14-days mortality were identified in the univariate analysis as shown in Table 5.

**Table 5:** Univariate analysis of the risk factors associated with 30-day mortality in ICU patients with BSI.

Variable	Died N = 531, (%)	Survived N = 520, (%)	Odd ratio	95% Confidence interval	P-value
Gender					
Female Male	239 (45.0%) 292 (55.0%)	239 (46.0%) 281 (54.0%)	0.98	0.77–1.24	0.8720
Age group					
18–64 65 and more	237 (44.6%) 294 (55.4%)	322 (61.9%) 198 (38.1%)	1.99	1.57–2.53	<0.0001∗
BMI					
<30 ≥30	247/355 (69.6%) 108/355 (30.4%)	281/418 (67.2%) 137/418 (32.8%)	0.922	0.68–1.24	0.5962
Smoking					
Yes	81 (15.2%)	93 (17.9%)	0.74	0.54–1.01	0.7409
Co-morbidities					
HTN CKD DM Pulmonary disease ESRD Solid tumor CVD MI AF CHF	324 (61.0%) 52 (9.8%) 253 (47.7%) 42 (7.9%) 26 (4.9%) 117 (22.0%) 87 (16.4%) 97 (18.3%) 38 (7.2%) 75 (14.1%)	260 (50.0%) 29 (5.6%) 228 (43.8%) 22 (4.2%) 48 (9.2%) 37 (7.1%) 64 (12.3%) 79 (15.2%) 29 (5.6%) 44 (8.5%)	1.50 1.70 1.13 1.71 1.81 3.72 1.35 1.11 1.19 1.79	1.17–1.89 1.04–2.80 0.98–1.43 1.04–2.80 1.13–2.95 2.57–5.46 0.97–1.89 0.82–1.52 0.73–1.94 1.23–2.62	0.0010∗ 0.0358∗ 0.2846 0.0358∗ 0.0132∗ <0.0001∗ 0.0724 0.4970 0.4700 0.0020∗
Number of co-morbidities					
0 1–4 ≥5	41 (7.7%) 426 (80.2%) 64 (12.1%)	103 (19.8%) 379 (72.9%) 38 (7.3%)	2.50 3.50	1.74–3.63 2.10–5.94	<0.0001∗ <0.0001∗
Mechanical ventilation					
Yes	44 (8.3%)	40 (7.7%)	1.17	0.75–1.83	0.4831
Prior catheterization					
Yes	44 (8.3%)	48 (9.2%)	0.98	0.65–1.49	0.9643
Previous hospitalization (90 days hospitalization)					
Yes	204 (38.4%)	175 (33.7%)	1.23	0.96–1.58	0.0957
Hospital transfer					
Yes	98 (18.5%)	133 (25.6%)	0.67	0.50–0.89	0.0064∗
Acquisition site					
Nosocomial Healthcare-associated Community-acquired	299 (56.3%) 67 (12.6%) 165 (31.1%)	239 (46.0%) 74 (14.2%) 207 (39.8%)	1.64 1.62	0.93–1.93 1.25–2.10	0.3288 0.0002∗
Source of infection					
Respiratory Genitourinary Line related GI SSTI CNS	218 (41.1%) 92 (17.3%) 13 (2.5%) 44 (8.3%) 18 (3.4%) 33 (6.2%)	142 (27.3%) 99 (19.0%) 34 (6.5%) 36 (6.9%) 51 (9.8%) 35 (6.7%)	1.73 0.93 0.44 1.10 0.31 0.86	1.35–2.21 0.69–1.26 0.24–0.79 0.72–1.71 0.17–0.52 0.53–1.39	<0.0001∗ 0.6528 0.0056∗ 0.6381 <0.0001∗ 0.5444
Vasopressor use					
Yes	263 (49.5%)	133 (25.6%)	2.91	2.26–3.76	<0.0001∗
Blood transfusion					
Yes	59 (11.1%)	73 (14.0%)	0.77	0.54–1.09	0.1477
Pathogens from other body sites					
Yes	350 (65.9%)	303 (58.3%)	1.52	1.19–1.93	0.0007∗
Type of antimicrobial coverage					
Monotherapy Dual-therapy Triple therapy or more	46 (8.7%) 122 (23.0%) 363 (68.4%)	107 (20.6%) 126 (24.2%) 287 (55.2%)	2.25 3.04	1.54–3.29 2.20–4.23	<0.0001∗ <0.0001∗
Septic shock					
Yes	212 (39.9%)	68 (13.1%)	4.61	3.42–6.29	<0.0001∗
Renal failure					
Yes	53 (10.0%)	23 (4.4%)	2.61	1.62–4.34	<0.0001∗
TPN					
Yes	203 (38.2%)	130 (25.0%)	2.01	1.55–2.61	<0.0001∗
ICU stay (days)					
≤7 days >7 days	204 (38.4%) 327 (61.6%)	270 (51.9%) 250 (48.1%)	1.72	1.36–2.19	<0.0001∗
Appropriate empiric					
Yes	88/185 (47.6%)	97/166 (58.4%)	0.64	0.42–0.98	0.0400∗
Appropriate definitive					
Yes	95/137 (69.3%)	130/168 (77.4%)	0.72	0.44–1.21	0.2223

BMI = Body mass index, HTN = Hypertension, CKD = Chronic kidney disease, DM = Diabetes mellitus, ESRD = End stage renal disease, CVD = Cerebro-vascular disease, MI = Myocardial infarction, AF = Atrial fibrillation, CHF = Congestive heart failure, GI = Gastro-intestinal, SSTI = Skin and soft tissue infection, CNS = Central venous system, TPN = Total parenteral nutrition, ICU = Intensive care unit.

Of note, Pulmonary disease was observed to increase 30-day mortality risk (OR 1.71, p = 0.0358). Moreover, patients with healthcare-associated infections had a significantly higher mortality rate (OR: 1.62, p = 0.0002).

The multivariable analysis of 30-day mortality identified age (≥65 years), solid tumors, line-related infections, and septic shock as independent predictors. Odds ratios and p-values are presented in Table 6.

**Table 6:** Multivariable analysis of the risk factors of 30-day mortality in ICU patients with BSIs.

Factor	Odd ratio	95% Confedance interval	P-value
Age group			
18–64 (R)65 and more	2.1	1.5–2.5	<0.0001∗
Solid tumor	3.35	2.26–4.91	<0.0001∗
Line-related	0.28	0.14–0.57	<0.0001∗
Septic shock	4.51	3.28–6.25	<0.0001∗

## 4 Discussion

The current study is the first study in Jordan to investigate the patterns of antimicrobial use and the predictors of mortality among ICU patients in a tertiary care hospital. It is well known that antimicrobial use is a major drive for antimicrobial resistance. During the ICU stay, all study participants were treated with at least one antimicrobial agent; more than half were treated with three and more antimicrobial agents.

In this current investigation, piperacillin/tazobactam teicoplanin, meropenem, levofloxacin, vancomycin, and imipenem were the most commonly used antimicrobials. This was similar to previous studies conducted on ICU patients [15, 16, 17]. Moreover, a 33-month surveillance study in Saudi Arabia reported that the most consumed antimicrobial agents in ICU were carbapenems, piperacillin/tazobactam, vancomycin, and colistin [18]. The selection of antimicrobial agents in patients in ICU patients can be attributed to the degree of ASP implementation in each country and institution, types and severity of the infections, and the availability of selected antimicrobials in the hospital’s formulary.

Therefore, the implementation of institution-specific guidelines of ASP will increase the appropriate antimicrobial utilization, increase the use of antibiotics with a narrower spectrum of activity, and shorter duration of therapy [19].

It is important to emphasize that the administration of broad-spectrum therapy is not always necessarily recommended and not as important as administering antimicrobial therapy actively against the most likely pathogens. Special attention should be conducted to each patient's risk factors and the most likely pathogen based on the infection sources of BSI before selecting the appropriate antimicrobial therapy.

This study reported pathogen distribution among ICU patients with BSI. Regarding gram-negative bacteria, the most frequent pathogens in this study were *Escherichia coli*, followed by *Klebsiella pneumonia, Acinetobacter baumannii, and Pseudomonas aeruginosa.*

Moreover, the most common gram-positive bacteria isolated from our patients were *MRSA,* followed by *MSSA*, *Enterococcus faecalis, Enterococcus faecium*, and *Streptococcus pneumonia*. These findings were similar to previous studies in countries worldwide [20, 21, 22, 23, 24, 25, 26, 27, 28].

In this retrospective analysis, the 14-day all-cause mortality was 47.9%, and the 30-day mortality was 50.5%. Similar findings were reported in a systematic review and meta-analysis in Europe and North America, where the overall ICU mortality increased from 37.3% to 51.9% when the septic shock was diagnosed [29]. However, other studies showed a lower mortality rate. For example, in one study in the US, the overall 30-day mortality in the ICU setting was 36.7% [30] whereas the overall mortality in another study in India was 28% [15]. A prospective nationwide surveillance study in the United States reported that the overall crude mortality ranged from 26% to 48% according to different infected pathogens in the ICU [31]. The implementation of ASP can be used to reduce the mortality rate; that was shown by Lindsay et al in his systematic review where the mortality rate was not changed with ASP applying the audit and feedback approach [32].

In the present study, several variables were identified to be associated with 14-day and 30-day mortality in patients BSIs; advanced age, presence of co-morbidities, nosocomial infections or healthcare-associated infections, length of ICU stay, respiratory tract infections, receiving vasopressor during a hospital stay, concurrent positive culture other than blood with BSI, receiving dual or more antimicrobial therapy, those who were complicated with septic shock or renal failure, receiving TPN nutrition and inappropriate empiric antimicrobial therapy. These findings come in agreement with previous literature around the world [16, 30, 31, 33]. As many of these factors are not modifiable, the approropiatness empirical therapy can play an essential role in mortality in this group of patients. Continuous review of hospital antibiograms, surveillance of antimicrobial use and resistance patterns, and teaching of hospital staff can guide the medical team to proper empirical therapy [34, 35].

In terms of MDR, this study showed a high prevalence of MDR phenotypes where almost three-quarters of our ICU patients were infected with MDR isolates. The higher rates of MDR in our institution require a profound effort of ASP teams in the selection of antimicrobial agents to provide an adequate appropriate empirical treatment which added to the challenges of the ASP team.

This study had a few limitations. First, it was a retrospective design that might pose hidden biases. There were limitations in data accessibility and availability, such as the severity of illness score (for example, APACHE II score), which was not incorporated because of unavailable data. Second, our study was a single-center, and the results may not be applied to other settings. Third, medical records did not identify obvious reasons for the delay in antimicrobial therapy. Finally, mortality in this study is crude mortality which may be affected by the patient's medical condition and severity of illness rather than the attributable mortality of BSI.

## 5 Conclusions

The present study investigated the types of antimicrobial use and mortality among ICU patients in a Jordanian tertiary care hospital. The administration of antimicrobials among ICU patients was highly based on a combination of three or more agents covering a broad spectrum of microorganisms. The mortality rate was high among patients who are believed to be given inappropriate empirical therapy. Therefore, we recommend that the hospital ASP protocol be re-evaluated for ICU patients. Several factors associated with mortality; advanced age, presence of co-morbidities, nosocomial infections or healthcare-associated infections, length of ICU stay, respiratory tract infections, receiving vasopressor during a hospital stay, concurrent positive culture other than blood with BSI, receiving dual or multiple antimicrobial therapies, septic shock or renal failure, TPN nutrition and inappropriate empiric antimicrobial therapy. Therefore, we recommend that hospital policies should apply the ASP practices and infection control.

## Declarations

### Author contribution statement

Mera A. Ababneh, Mohammad Al Domi and Abeer M Rababa'h: Conceived and designed the experiments; Performed the experiments; Analyzed and interpreted the data; Wrote the paper.

### Funding statement

This work was supported by Deanship of Research at Jordan University of Science and Technology, Irbid, Jordan (Grant #: 20200248).

### Data availability statement

Data will be made available on request.

### Declaration of interests statement

The authors declare no conflict of interest.

### Additional information

No additional information is available for this paper.
